# Detection and Classification of Stroke Gaits by Deep Neural Networks Employing Inertial Measurement Units

**DOI:** 10.3390/s21051864

**Published:** 2021-03-07

**Authors:** Fu-Cheng Wang, Szu-Fu Chen, Chin-Hsien Lin, Chih-Jen Shih, Ang-Chieh Lin, Wei Yuan, You-Chi Li, Tien-Yun Kuo

**Affiliations:** 1 Department of Mechanical Engineering, National Taiwan University, Taipei 106, Taiwan; r09522828@ntu.edu.tw (C.-J.S.); r06522835@ntu.edu.tw (W.Y.); r07522829@ntu.edu.tw (Y.-C.L.); r08522836@ntu.edu.tw (T.-Y.K.); 2 Department of Physical Medicine and Rehabilitation, Cheng Hsin General Hospital, Taipei 112, Taiwan; szufuchen@yahoo.com.tw (S.-F.C.); lovephysics25@gmail.com (A.-C.L.); 3 Department of Physiology and Biophysics, National Defense Medical Center, Taipei 114, Taiwan; 4 Department of Neurology, National Taiwan University Hospital, Taipei 100, Taiwan; chlin@ntu.edu.tw

**Keywords:** gait recognition, deep learning, neural network, stroke gait, IMU (inertial measurement unit)

## Abstract

This paper develops Deep Neural Network (DNN) models that can recognize stroke gaits. Stroke patients usually suffer from partial disability and develop abnormal gaits that can vary widely and need targeted treatments. Evaluation of gait patterns is crucial for clinical experts to make decisions about the medication and rehabilitation strategies for the stroke patients. However, the evaluation is often subjective, and different clinicians might have different diagnoses of stroke gait patterns. In addition, some patients may present with mixed neurological gaits. Therefore, we apply artificial intelligence techniques to detect stroke gaits and to classify abnormal gait patterns. First, we collect clinical gait data from eight stroke patients and seven healthy subjects. We then apply these data to develop DNN models that can detect stroke gaits. Finally, we classify four common gait abnormalities seen in stroke patients. The developed models achieve an average accuracy of 99.35% in detecting the stroke gaits and an average accuracy of 97.31% in classifying the gait abnormality. Based on the results, the developed DNN models could help therapists or physicians to diagnose different abnormal gaits and to apply suitable rehabilitation strategies for stroke patients.

## 1. Introduction

Stroke is a common medical emergency with a high mortality rate, ranking second among the leading causes of death in the last 15 years [1]. However, the patients who are fortunate enough to survive stroke events usually suffer from partial disability and inconvenience in their daily lives. Therefore, post-stroke patients usually require long-term healthcare and rehabilitation. At present, stroke costs are about 34 billion US dollars per year in the USA [2]. On average, each stroke patient spends about 60,000 US dollars per year, with 30% of those costs expended on rehabilitation and medical care [3]. The purpose of rehabilitation is to help these patients recover their ability for independent living, particularly walking on their own.

Hemiplegia is one of the most common impairments after stroke and contributes significantly to walking impairments [4]. The hemiplegic gait patterns include decreased knee flexion and ankle dorsiflexion during swing [5]. Approximately two thirds of stroke survivors with initial paralysis of the leg can eventually walk with or without assistance [6]. However, many individuals still exhibit considerable gait impairments and cannot achieve the walking dexterity that enable them to perform all their daily activities. Stroke survivors normally develop abnormal gaits, such as longer swing phases and decreased stance phases on the paretic side. Because different and diverse gait problems are encountered in stroke survivors, it is important to determine each individual’s gait abnormalities during the golden time window of rehabilitation to develop appropriate training strategies and improve patients’ functional outcomes.

Unfortunately, most of the gait training programs have been based mainly on clinical assessments, which may be influenced by subjective decisions and therapists’ experiences. For this reason, many researchers have attempted to identify the walking pattern in objective manners. For example, Zhao et al. [7] proposed the rule-based algorithms to identify several gait events. Wang et al. [8] developed algorithms to detect three gait events in real time. Knutsson and Richards [9] used electromyogram signals to identify three types of abnormal muscle activation patterns in post-stroke patients. Wong et al. [10] applied load sensors to analyze the foot contact pattern when evaluating walking ability in patients with hemiplegic stroke. Some studies have also applied machine learning techniques to develop gait classification models. For instance, Wahid et al. [11] measured spatial-temporal gait signals for feature extraction and proposed a classification model to diagnose Parkinson disease (PD); their model achieved an accuracy of 92.6% after normalizing gait data using a multiple regression approach. Daliri [12], who analyzed gait signals derived from ground reaction forces, applied support vector machines to diagnose subjects with PD and achieved an accuracy of 91.2%. Similarly, Dolatabadi et al. [13] applied two machine learning methods and discriminated normal and pathological gait patterns with an accuracy of more than 90%. Li [14] used the dynamic time warping algorithm, sample entropy method, and empirical mode decomposition-based stability index to analyze the symmetry, regularity, and stability of post-stroke hemiparetic gaits. They then applied the k-nearest-neighbor classifier to distinguish stroke gaits and achieved an area-under-the-curve value of 0.94. However, no research has yet been conducted on the classification of stroke gait patterns. Therefore, the aim of this paper is to develop models that can recognize stroke gaits.

The gait abnormalities in post-stroke patients vary, and each needs specific rehabilitation strategies. For example, the following are four common gait abnormalities seen in post-stroke patients:

(1) The drop-foot gait [15,16,17]: Patients develop a drop foot gait because their weakness or paralysis limits their ability to raise the front part of the foot, so that their toes are dragged when walking. This abnormal gait can slow walking speed and increased risk of falls.
(2) The circumduction gait [18,19,20,21,22,23]: This gait is also known as the neurological or hemiplegia gait. The knee and hip movements are insufficient to allow the foot to clear the ground, so the patients adopt an abnormal walking pattern by taking the leg away from the body and swinging the leg forward in a semicircular fashion when walking.
(3) The hip hiking gait [18,24,25]: This gait is defined as a frontal plane elevation of the ipsilateral side of the pelvis to achieve foot clearance. Both hip hiking and circumduction are secondary gait deviations used to achieve ground clearance during the paretic swing phase.
(4) The back knee gait [26,27,28,29]: This gait is also known as genu recurvatum, which is defined as full extension or hyperextension of the knee in the stance phase [27]. Genu recurvatum can lead to functional mobility limitations and early degenerative changes of knee joint due to progressive knee hyperextension [28].

The abnormal gaits in post-stroke patients not only increase energy consumption but they also reduce walking efficiency, causing difficulties in ambulation and increasing the risk of falling. The abnormal gaits can also place extra pressure on the joints, cause damage to joints or ligaments, and even affect the patient’s mental state and quality of life. Hence, evaluation and identification of gait abnormalities are important for developing appropriate training strategies for rehabilitation. In current clinical practice, gait pattern diagnosis mainly relies on the experience of clinicians or physical therapists to make judgments, and there is no objective diagnostic standard. Different clinicians or therapists might have different diagnoses of stroke gait patterns. In addition, some patients may present with mixed neurological gaits, for example, hemiplegic gait combined with foot inversion gait due to increased muscle tone or some patients may combine with drop foot because of receiving over-dose botulinum toxin treatment for legs spasticity. Therefore, many researchers have attempted to identify gait events [7,8] and gait abnormalities [9,10]. Machine learning techniques are also applied to improve the identification performance [11,12,13,14]. However, no research has yet been conducted on the classification of stroke gaits. Hence, in this paper we apply deep learning technologies to detect and classify stroke gaits as an aid to diagnosis and for application of appropriate rehabilitation methods for stroke patients. In this study, the stroke gait patterns were diagnosed by two physical therapists who were more than 15 years qualified, with at least 10 years of daily experience working with patients with stroke. Then we apply the clinical data to develop Deep Neural Network (DNN) models to assist physical therapists or physicians for more optimizing the diagnosis of different stroke gaits, especially for those with mixed neurological gait problems.

The paper is arranged as follows: Section 2 introduces the experiments for the collection of gait data. We applied inertial measurement units (IMUs) to acquire the gait information. Section 3 develops a deep-learning model for recognizing stroke gaits. Section 4 describes the model training processes and validation. We also test the developed models by applying a public dataset. Based on the results, we discuss the performance, limitation, and future development of the developed model in Section 5. Finally, we draw conclusions in Section 6.

## 2. Collection and Processing

This section describes the experiments conducted to collect gait data. We invited stroke patients and healthy subjects to conduct walking tests. Their gaits were measured and applied to develop a DNN model that can identify and classify the stroke gaits.

First, we recruited eight post-stroke patients; their data are illustrated in Table 1. The following criteria were applied when selecting the test subjects: (1) the Brunnstrom Stage (BS) [30] on the lower extremity was stage 3–5; (2) the Functional Ambulation Category (FAC) [31] was stage 3–5; (3) the Mini-Mental State Examination (MMSE) [32] score was higher than 24; (4) subjects could walk ten meters indoors with or without aid devices, and (5) subjects could stand up on their own using a handrail and aids. Second, we also recruited seven healthy subjects as the normal reference group; their data are illustrated in Table 2. All test subjects signed informed consent forms approved by the Human Subject Research Ethics Committee of Institutional Review Board (IRB) [33], as shown in Appendix A.

**Figure 1:**
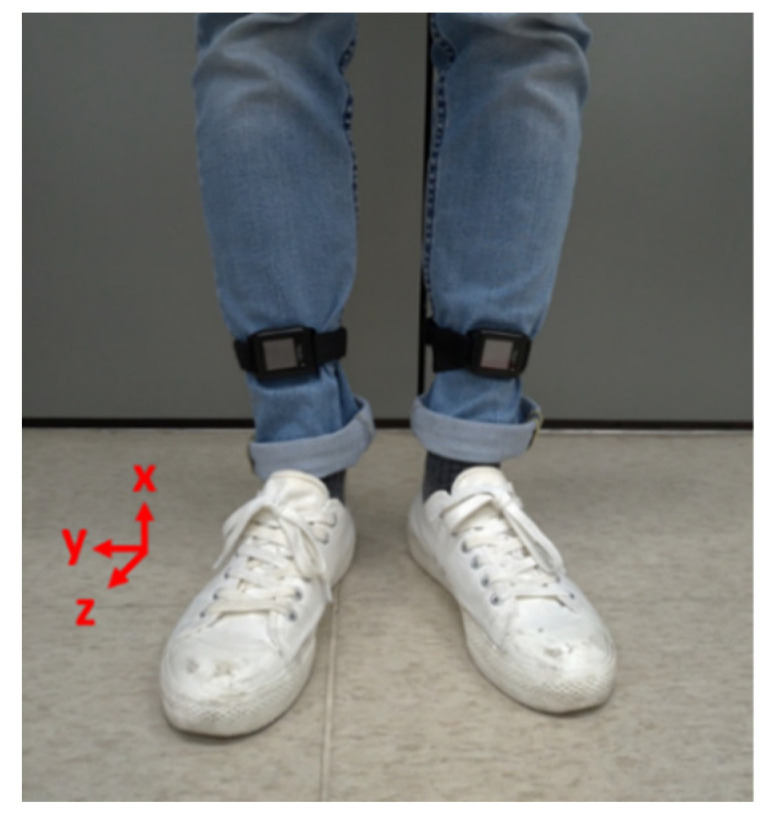
Gait measurements by two inertial measurement units (IMUs).

Wearable sensor technologies have been frequently applied to gait analyses. For example, Diaz et al. [34] surveyed the applications of wearable sensor technologies in analyzing the gait, balance, and range of motion research. Nguyen et al. [35] applied an IMU-based system to develop deep convolutional neural network models for distinguish subjects with foot structural abnormalities. In this paper, we applied the APDM OPAL system [36] to acquire the gait information. The OPAL system contains wearable IMUs with a sampling rate of 128 Hz and a resolution of 17.5 bits. Each IMU has a size of about 44 mm×40 mm×14 mm and weighs less than 25 gm. Two IMUs were attached to the subjects’ shanks, as shown in Figure 1. Each IMU consists of a 3-axis accelerometer, a 3-axis gyroscope, and a 3-axis magnetometer. The maximum measurement ranges of the accelerometer, the gyroscope, and the magnetometer are ±200 g, ±200 deg/s, and ±8 Gauss, respectively. This IMU device can detect a subject’s kinematic data with a highest sampling rate of 128 Hz. In the experiment, all subjects were required to complete walking tests at their most comfortable pace.

**Figure 2:**
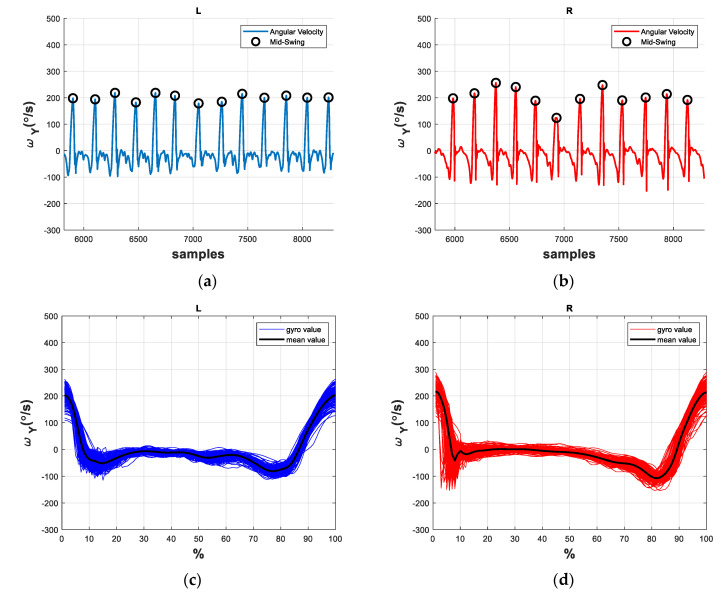
The angular velocities and gait patterns of a stroke subject P8. (**a**) angular velocity of the left leg; (**b**) angular velocity of the right (paretic) leg; (**c**) gait cycles of the left leg; (**d**) gait cycles of the right (paretic) leg.

We applied the angular velocity of the shank on the sagittal plane [37], which is the mediolateral axis (y-axis) in Figure 1, to develop the DNN model. For example, the angular velocities $\omega_{y}$ of patient P8 are shown in Figure 2a,b, where L and R represents the left leg and the right leg, respectively. Each gait cycle contains the following three important gait events [38]: (1) Mid-swing: when the angular velocity achieves its maximum in the gait cycle; (2) Heel strike: when the heel touches the ground, where the angular velocity has the first negative trough after the mid-swing; (3) Toe off: when the toes leave the ground. Because these gait events can be evaluated by the angular velocities [37], we can mark the mid-swing points of each gait and divided the measured data into individual gait cycles, as shown in Figure 2c,d. Note that we split the gait cycles by the mid-swing in that labelling the mid-swing is much easier and more direct than labelling the heel-strike [8]. The model development will be similar if the gait cycles are partitioned by the heel-strike or the toe-off. We then normalized the gait data by dividing it into one hundred points for each gait cycle; this was done because each subject had a different walking speed and the data length for each gait cycle was not the same.

**Figure 3:**
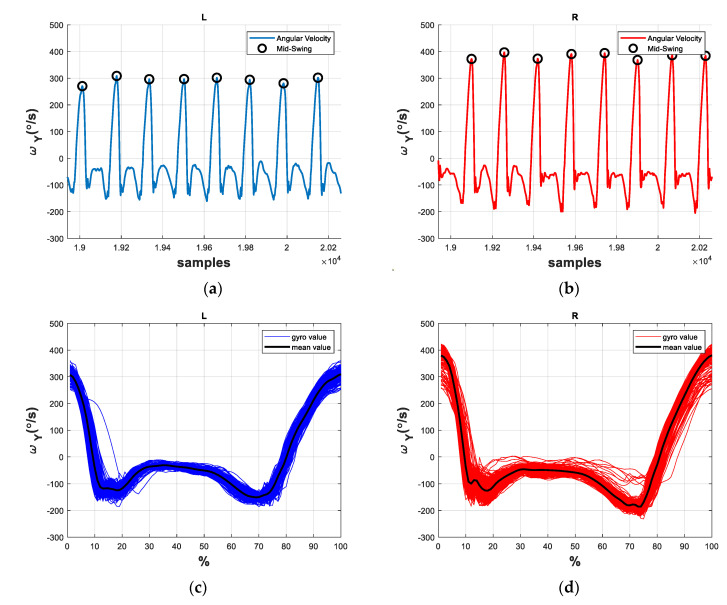
The angular velocities and gait patterns of a healthy subject H7. (**a**) Angular velocity of the left leg; (**b**) angular velocity of the right leg; (**c**) gait cycles of the left leg; (**d**) gait cycles of the right leg.

By contrast, we illustrate the angular velocities $\omega_{y}$ of the healthy subject H7 in Figure 3, where the healthy subject’s gaits tend to be smoother than the stroke patient’s gaits shown in Figure 2. We further compare the gait cycles of other stroke patients and healthy subjects, as shown in Appendix B, and find that the stroke subjects’ gaits have significantly more trembles and vibration, especially on the paretic side. Hence, it is not difficult to distinguish the stroke gaits from the healthy gaits [10,13,14]. Nevertheless, the classification of abnormal stroke gaits is challenging because each patient might develop various combination of gait abnormalities which simultaneously affect the gait patterns. Therefore, we propose a DNN model structure for detecting and classifying the stroke gaits.

We labelled the gait data from the stroke patients and the healthy subjects as the stroke gait (SG) and the normal gait (NG), respectively. The stroke gaits were further classified as the stroke gait with a drop foot (SGwDF), the stroke gait with circumduction (SGwC), the stroke gait with hip hiking (SGwHH), and the stroke gait with back knee (SGwBK), according to the therapists’ diagnoses. Because the patients might or might not have had these abnormal gaits, we set four labels to build a multi-label classification model, as shown in Table 3. For example, patient P5 had all four gait abnormalities on the right leg, while patient P6 had both the drop foot gait and the back knee gait on the left leg. Conversely, Patient P7 had only the hip hiking gait on the left leg. We applied the gait data from Table 3 to establish a gait dataset with 4037 gaits, including 2037 stroke gaits and 2000 normal gaits, as illustrated in Appendix C.

## 3. Deep Neural Network Model

This section develops a DNN model to recognize stroke gaits. We applied the measured gait data to develop a DNN model that can identify and classify the stroke gaits.

**Figure 4:**
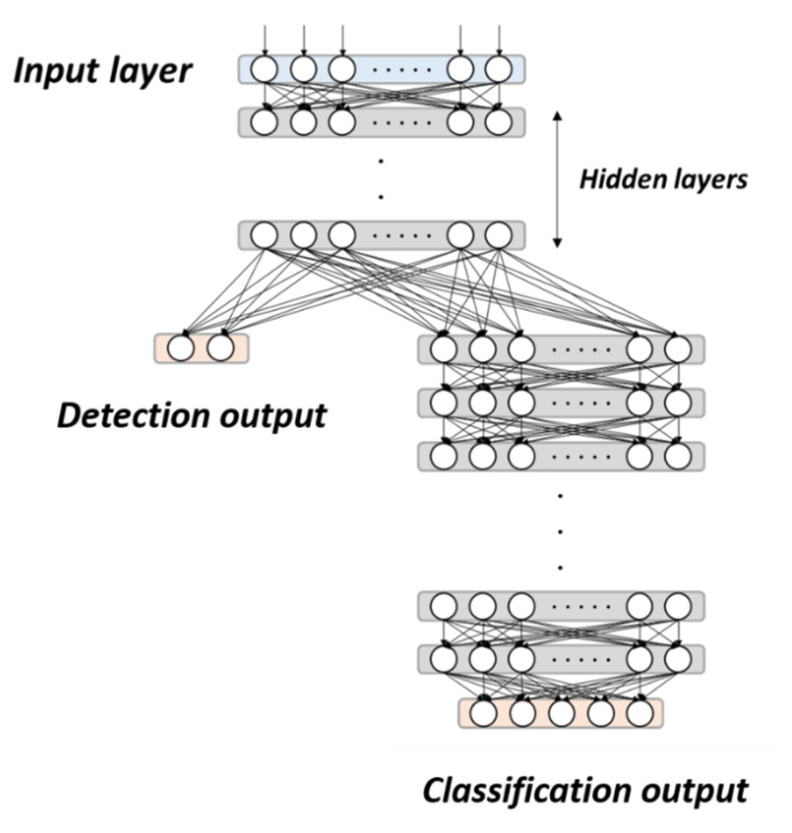
Architecture of the deep neural network (DNN) model.

We applied the normalized gait data to build a multi-output gait recognition model. The model architecture is shown in Figure 4 and includes the detection part and the classification part. The detection part first judges whether the input gait is a normal gait or a stroke gait. It contains the input layer, six hidden layers, and the detection output. Each fully connected layer has 100 neurons. The numbers of hidden layers and neurons were chosen by iterative tests. Because the DNN model is highly nonlinear, using more layers and neurons might result in similar accuracy but greatly increase the computing loads. For example, the computing time was increased by about 75% when using ten layers. The detection output has two neurons to label the gait as NG or SG, marked as [1, 0] or [0, 1], respectively. The classification part then analyzes the stroke gait if the detection output indicates SG. It contains ten hidden layers and the classification output, where each hidden layer has 100 neurons. The classification output has five neurons to classify stroke gaits as SG, SGwDF, SGwC, SGwHH, and SGwBK. For example, the stroke gaits with drop foot and hip hiking are labelled as [1, 1, 0, 1, 0], while the stroke gaits with all four gait abnormalities are labelled as [1, 1, 1, 1, 1].

**Figure 5:**
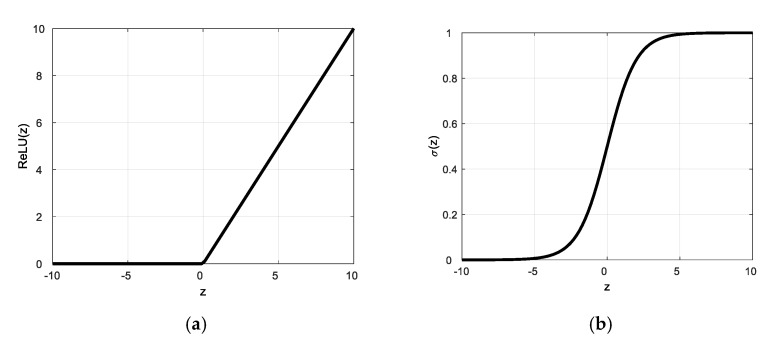
The activation functions. (**a**) the rectified linear unit (ReLU) function; (**b**) the sigmoid function.

We applied the following functions to develop the DNN model:

(1) The Activation Function: The neural network applied nonlinear activation functions in neurons. We selected the rectified linear unit (ReLU) [39], as shown in Figure 5a, as the activation function for the hidden layers:
  (1) ReLU(z)=max(0,z)
  where z is the neuron input and ReLU(z) is the neuron output. This function can effectively overcome the vanishing gradient problem when updating the model parameters by back propagation [40]. The neural network would not continue training when the learning gradient is small. Moreover, the computing load is reduced because the function judges whether the input is greater than 0. That is, the ReLU function is a complete transfer for positive gradients with a derivative of 1. If the input z<0, then ReLU(z) = 0 and this neuron is directly deleted, thereby achieving a reduction in neurons and allowing rapid convergence.
  Conversely, we selected the following sigmoid function [41] as the activation function of the output layers:
  (2) $$\sigma \left(z\right)=\frac{1}{1+e^{-z}}$$
  where z is the neuron input and σ(z) is the neuron output. The sigmoid function converts a scalar number to [0, 1], as shown in Figure 5b. If σ(z) is greater than a threshold of 0.5, it is considered to belong to the labelled category. The sigmoid function is the optimized fitting function of the binary classification problem, where its output corresponds to the loss function mentioned in the next section. Because the probability of each label is independent, the sigmoid function is usually used as the activation function of the output layer for multi-label classification.
(2) The Loss Function: The loss function is applied to evaluate how well the algorithms interpret the given data. This function evaluates the loss of the model and updates the weights to reduce the loss on the next evaluation. We applied the following cross-entropy [42] as the loss function:
  (3) $$C\left(y,\hat{y}\right)=-\frac{1}{n}\sum_{i=1}^{n}{\hat{y}}_{i}\cdot \log(y_{i})+(1-{\hat{y}}_{i})\cdot \log(1-y_{i})$$
  where $y_{i}$ is the distribution of the true output and ${\hat{y}}_{i}$ is the distribution of the predicted output. Cross entropy can measure and quantify the similarity between $y_{i}$ and ${\hat{y}}_{i}$. In addition, it can help to avoid learning rate decreases in the gradient descent [43] by simultaneously applying the cross-entropy as the loss function and the sigmoid function as the activation function to the output layer.
(3) The Optimizer: We selected Adam [44] as the optimizer of the DNN model. Adam is an adaptive learning rate optimization algorithm designed specifically for training DNNs, because it combines the advantages of Adagrad [45] and RMSprop [46] by calculating the gradients and updating the weights [43].

## 4. Model Training and Validation

**Figure 6:**
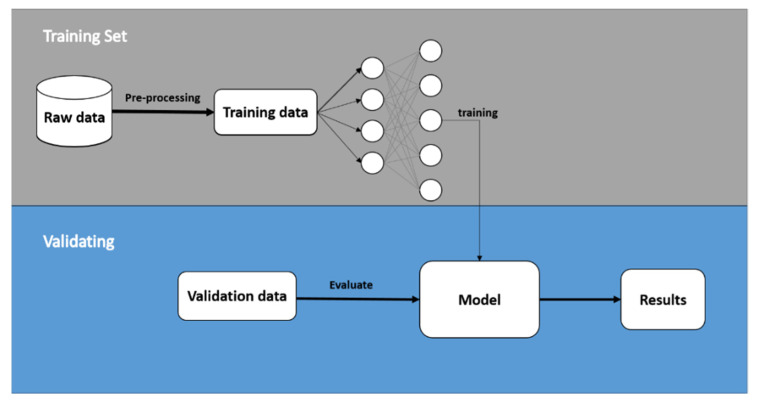
The model training and validating procedures.

This section introduces the model training process and model validation. We applied the k-fold cross-validation test [47] to evaluate the performance of the model. In this paper, we set k = 4 by dividing all classes of gait data into four parts (Fold 1, Fold 2, Fold 3, and Fold 4), and we then arranged them randomly for training and validating. Each training took three of the four folds as a training dataset and used the remaining fold as validation. Figure 6 shows the training and validating flow chart, where the 4-fold cross-validation was repeated four times.

In the training process, 500 samples were selected for each model training (batch size = 500) to update the weights. The training data passed through the layers of the model with present parameters and the ReLU function. Then, the obtained evaluation output was compared with the ground truth label by clinical experts to calculate the corresponding model loss by the loss function and to update model parameters by back propagation. This training process was repeated sixty times (Epochs = 60), where the model parameters were optimized and the model loss was minimized by repeated training. The phenomena of overfitting and excessive time in the training process were avoided by adding Dropout [48], with a dropout rate of 0.2, to each fully connected layer in the classification part of the model. This gave each neuron a probability of 20% of being deleted. Finally, the remaining fold was inputted to the trained models to verify the correction of these models in the validation process.

The correction of a model is frequently quantitatively indicated by the confusion matrix, as illustrated in Table 4. Based on Table 4, the following indicators are frequently applied to evaluate the quality of model training [49]:

(4) $$Accuracy=\frac{\mathrm{TP}+\mathrm{TN}}{\mathrm{TP}+\mathrm{FP}+\mathrm{FN}+\mathrm{TN}}$$

(5) $$Precision=\frac{\mathrm{TP}}{\mathrm{TP}+\mathrm{FP}}$$

(6) $$Recall=\frac{\mathrm{TP}}{\mathrm{TP}+\mathrm{FN}}$$

(7) $$F1-score=\frac{2\times Precision\times Recall}{Precision+Recall}$$

where *Accuracy* is the most intuitive indicator, although it might be invalid in some cases [49]. In this paper, we apply *Accuracy* and *F1-score* to demonstrate the quality of the developed DNN model.

The confusion matrix of the detection layer is shown in Table 5, where model *i* applied all gait data except Fold *i* for training and used the gait data of Fold *i* for validation. The results show that the model can successfully identify the stroke gaits with only few errors on the FP and FN terms. The confusion matrix of the classification layer is shown in Table 6, where P and N represent positive and negative, respectively. We independently list the output neurons and observe that some errors occur in classifying the four gait abnormalities: drop foot, circumduction, hip hiking, and back knee gaits. The overall test results are shown in Table 7. The detection layer achieves an average accuracy of 99.35% and an average F1-score of 0.9935 in detecting the stroke gaits, while the classification layer achieves an average accuracy of 97.31% and an average F1-score of 0.9662 in classifying the abnormal stroke gaits.

Gait is a symmetrical and rhythmic periodic motion that can be disrupted by stroke. Abnormal stroke gaits can decrease the efficiency of walking; therefore, the identification of gait abnormalities and the development of appropriate training strategies for rehabilitation are very important. From Table 7, the developed DNN models are deemed effective in detecting and classifying stroke gaits. However, the classification model is not as good as the detection model. One possible reason is that the number of stroke gaits in the dataset is insufficient; hence, the number of samples for SGwDF, SGwC, SGwHH, and SGwBK is not representative. That is, the accuracy of the classification can be further improved by adding additional abnormal gaits. In the future, we can collect more abnormal gait data to further improve the efficiency of the model.

We also applied the public dataset Physical Activity Monitoring Data Set (PAMAP2) [50], available in the UCI machine learning repository [51], to evaluate the developed model. This dataset consists of nine healthy subjects (one female and eight males) who wore IMU devices and conducted 12 different activity tests, including standing, sitting, and walking. We applied the angular velocity of the shank in the walking activities as the input data to the four DNN models. Because there is no public dataset for stroke gaits, we invited two stroke patients to conduct experiments and measured their gaits to test the developed models. The data of these two stroke subjects are illustrated in Appendix D and their gait data is illustrated in Appendix C. The testing results are shown in Table 8, where the average accuracy is 99.34% and the average F1-score is 0.9939. That is, the developed DNN model is effective in detecting gaits from data in public domains.

## 5. Discussion

This study develops DNN models to recognize four common stroke gaits, including those with a drop foot, circumduction gait, those with hip hiking, and the stroke gait with back knee. Stroke patients usually suffer from partial disability and develop abnormal gaits that can vary significantly and need targeted rehabilitation strategies. Therefore, evaluation of gait patterns is crucial for clinical experts to make decisions on the medication and rehabilitation methods for the stroke patients. In current clinical practice, gait pattern diagnosis mainly relies on the experience of clinicians or physical therapists to make judgments, and there is no objective diagnostic standard. Different clinicians or therapists might have different diagnosis of stroke gait patterns. In addition, some patients may present with mixed neurological gaits. Therefore, many researchers have attempted to develop objective means for identifying gait events and gait abnormalities [7,8,9,10]. Some studies applied machine learning techniques to improve the identification performance [11,12,13,14]. However, no research has yet been conducted on the classification of stroke gaits. Hence, in this paper, we applied deep learning technologies to detect and classify stroke gaits as an aid to diagnosis and for application of appropriate rehabilitation methods for stroke patients.

In this study, we collected clinical gait data from eight stroke patients and seven healthy subjects. Their gait patterns were diagnosed by two physical therapists who were more than 15 years qualified, with at least 10 years of daily experience working with patients with stroke. Then, we apply the clinical data to develop DNN models to detect stroke gaits and to classify four common gait abnormalities seen in stroke patients. The developed models were shown to achieve high accuracies in detecting the stroke gaits and classifying the gait abnormality. Therefore, our DNN model could assist physical therapists or physicians for more optimizing the diagnosis of different stroke gaits and making decisions about the medication and rehabilitation strategies for the stroke patients. The limitation of this study is the number of enrolled patients is relatively small. A future large-scale study enrolling more patients with stroke is needed to validate the effectiveness of our established DNN structure.

## 6. Conclusions

This paper has developed DNN models that can detect stroke gaits and classify gait abnormalities. First, we collected clinical gait data from eight stroke patients and seven healthy subjects. The stroke gaits were further analyzed to indicate four common abnormal gaits in the stroke patients: the drop foot gait, the circumduction gait, the hip hiking gait, and the back knee gait. We applied IMUs to collect gait information from the stroke patients and healthy subjects. The obtained gait data were then analyzed to establish an expert dataset and to develop DNN models for recognizing the stroke gaits. The results showed that the developed DNN models achieved an average accuracy of 99.35% for detecting the stroke gaits and an average accuracy of 97.31% for classifying the gait abnormality. We also applied the PAMAP2 public dataset to the DNN models and achieved an average accuracy of 99.92% and an average F1-score of 0.9996. The effectiveness of the DNN structure could be further verified by recruiting more subjects. In the future, the proposed DNN model could help therapists to identify abnormal gaits and to apply suitable rehabilitation methods. The model can be further expanded in the future to identify more types of abnormal gaits, such as the Trendelenburg gait and the scissor gait, associated with other neurologic or musculoskeletal disorders.

## Figures and Tables

**Table 1:** Basic data of the stroke subjects.

Stroke Subject								
Subject	Gender	Age	Height (cm)	Weight (kg)	Paretic Side	MMSE (Score)	BS (Stage)	FAC (Stage)
P1	Male	51	174	66	Right	30	3	6
P2	Male	48	168	61	Right	28	3	6
P3	Female	61	161	56	Right	29	4	6
P4	Male	53	162	75	Left	29	3	6
P5	Male	52	173	81	Right	27	3	6
P6	Male	72	168	75	Left	29	5	6
P7	Male	64	158	61	Left	30	5	6
P8	Female	69	156	90	Right	30	4	6

**Table 2:** Basic data of the healthy subjects.

Healthy Subject				
Subject	Gender	Age	Height (cm)	Weight (kg)
H1	Male	24	185	85
H2	Male	24	178	70
H3	Male	25	170	63
H4	Male	25	164	70
H5	Male	24	172	75
H6	Male	26	172	76
H7	Male	23	166	62

**Table 3:** The labelled data.

Subject		Number of Gaits	NG	SG	SGwDF	SGwC	SGwHH	SGwBK
P1	left	50	0	1	0	0	0	0
	right	39	0	1	0	0	1	1
P2	left	68	0	1	0	0	0	0
	right	52	0	1	1	0	1	0
P3	left	92	0	1	0	0	0	0
	right	76	0	1	0	1	0	0
P4	left	187	0	1	0	1	1	1
	right	190	0	1	0	0	0	0
P5	left	169	0	1	0	0	0	0
	right	158	0	1	1	1	1	1
P6	left	158	0	1	1	0	0	1
	right	171	0	1	0	0	0	0
P7	left	139	0	1	0	0	1	0
	right	158	0	1	0	0	0	0
P8	left	155	0	1	0	0	0	0
	right	175	0	1	1	0	1	0
Healthy Subjects	left	1000	1	0	0	0	0	0
	right	1000	1	0	0	0	0	0

**Table 4:** The confusion matrix.

		Actual	
		Positive	Negative
**Predicted**	Positive	TP	FP
	Negative	FN	TN

TP: True Positive; FP: False Positive; FN: False Negative; TN: True Negative.

**Table 5:** Confusion matrix of the DNN models for stroke detection.

Actual			Normal Gait		Stroke Gait	
Predicted			Positive	Negative	Positive	Negative
Model 1	validation by Fold 1	Positive	496	3	497	5
		Negative	4	497	3	495
Model 2	validation by Fold 2	Positive	495	2	500	8
		Negative	5	498	0	492
Model 3	validation by Fold 3	Positive	498	2	497	2
		Negative	2	498	3	498
Model 4	validation by Fold 4	Positive	496	3	497	3
		Negative	4	497	3	497

**Table 6:** Confusion matrix of the DNN models for the classification of stroke gaits.

	Actual	Stroke Gait		Drop Foot		Circumduction		Hip Hiking		Back Knee	
Predicted		P	N	P	N	P	N	P	N	P	N
Model 1	P	497	5	77	5	77	0	154	10	105	8
	N	0	0	2	418	20	405	17	321	21	368
Model 2	P	500	8	96	7	98	11	178	14	112	5
	N	0	0	4	401	0	399	4	312	20	371
Model 3	P	497	2	101	4	113	2	197	5	131	8
	N	0	0	6	388	4	380	10	287	1	359
Model 4	P	497	3	89	7	98	9	168	13	123	4
	N	0	0	4	400	4	489	5	314	19	354

**Table 7:** Validation results of the DNN models.

	Detection		Classification	
	Accuracy	F1-Score	Accuracy	F1-Score
Model 1	0.9925	0.9925	0.9649	0.9539
Model 2	0.9925	0.9925	0.9717	0.9642
Model 3	0.9955	0.9955	0.9831	0.9802
Model 4	0.9935	0.9935	0.9728	0.9663
Average	0.9935	0.9935	0.9731	0.9662

**Table 8:** Gait detection using the data set Physical Activity Monitoring Data Set (PAMAP2) and two extra stroke patients.

	Actual	PAMAP2 Test				Sv1 and Sv2			
Predicted		P	N	Accuracy	F1-Score	P	N	Accuracy	F1-Score
Model 1	P	1005	0	1	1	219	0	0.9909	0.9909
	N	0	0			2	0		
Model 2	P	1004	0	0.9990	0.9995	219	0	0.9909	0.9909
	N	1	0			2	0		
Model 3	P	1005	0	1	1	217	0	0.9819	0.9841
	N	0	0			4	0		
Model 4	P	1003	0	0.9980	0.9990	218	0	0.9864	0.9864
	N	2	0			3	0		

**Figure A1:**
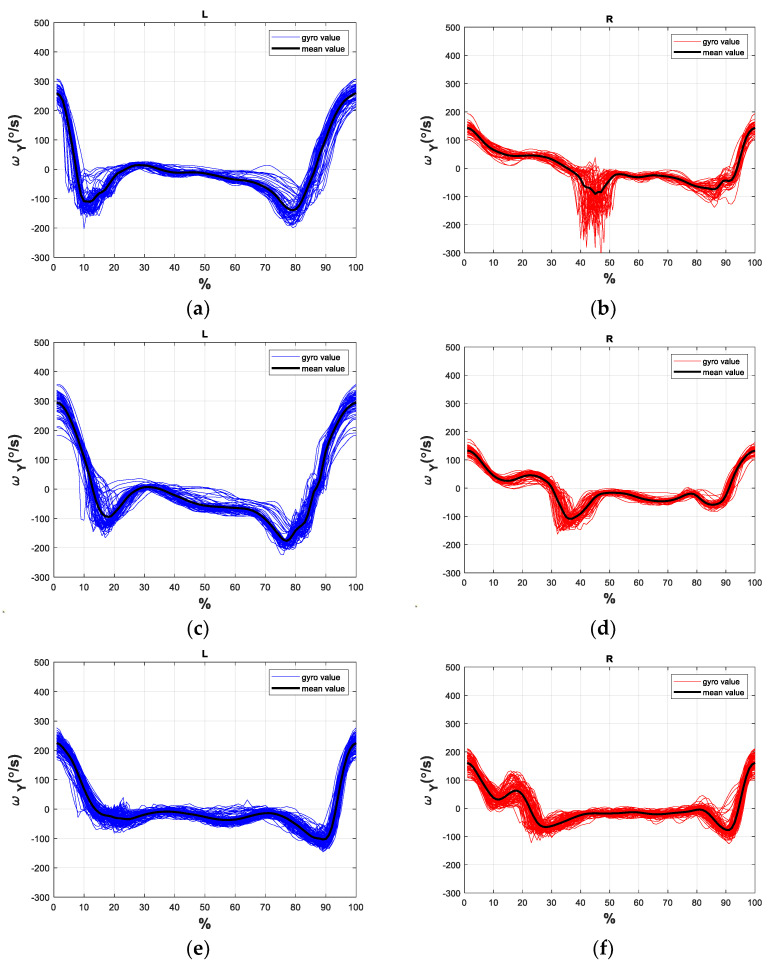

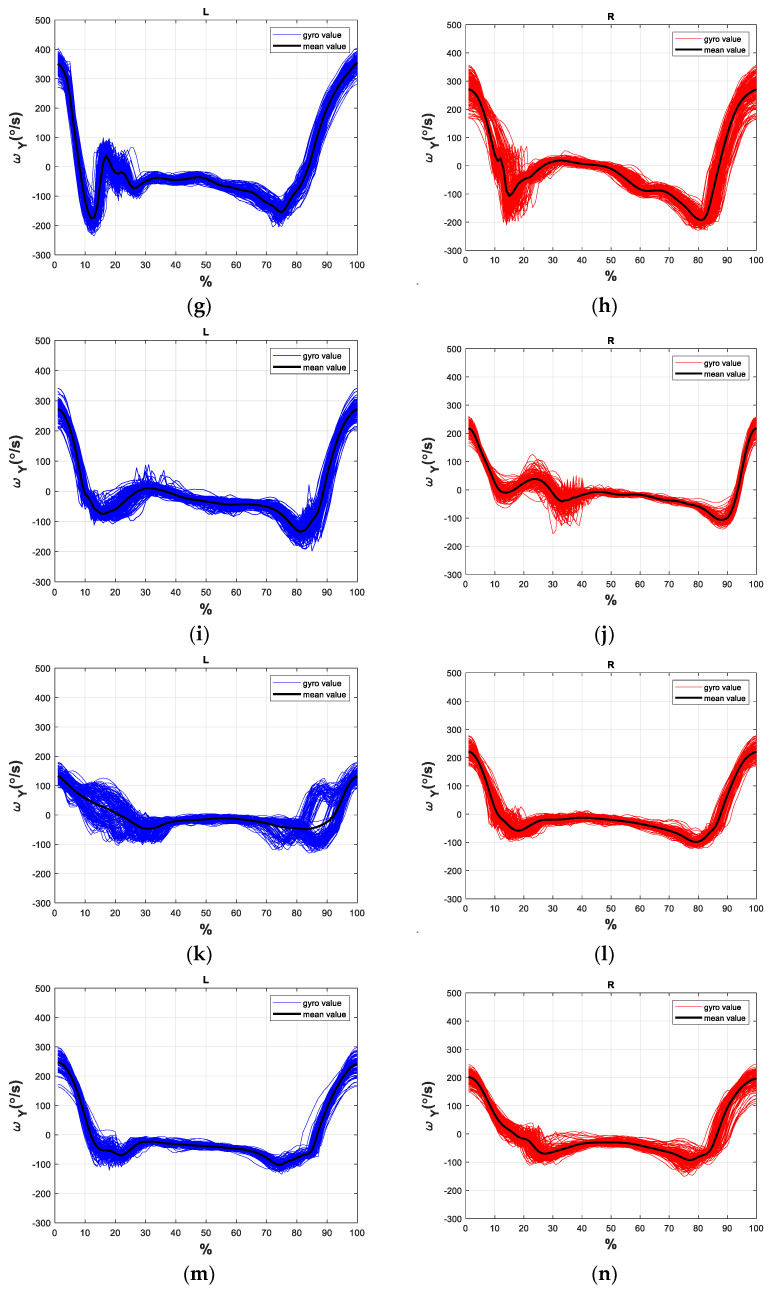
The gait patterns of stroke subjects. (**a**) left gait cycles of P1; (**b**) right gait cycles of P1; (**c**) left gait cycles of P2; (**d**) right gait cycles of P2; (**e**) left gait cycles of P3; (**f**) right gait cycles of P3; (**g**) left gait cycles of P4; (**h**) right gait cycles of P4; (**i**) left gait cycles of P5; (**j**) right gait cycles of P5; (**k**) left gait cycles of P6; (**l**) right gait cycles of P6; (**m**) left gait cycles of P7; (**n**) right gait cycles of P7.

**Figure A2:**
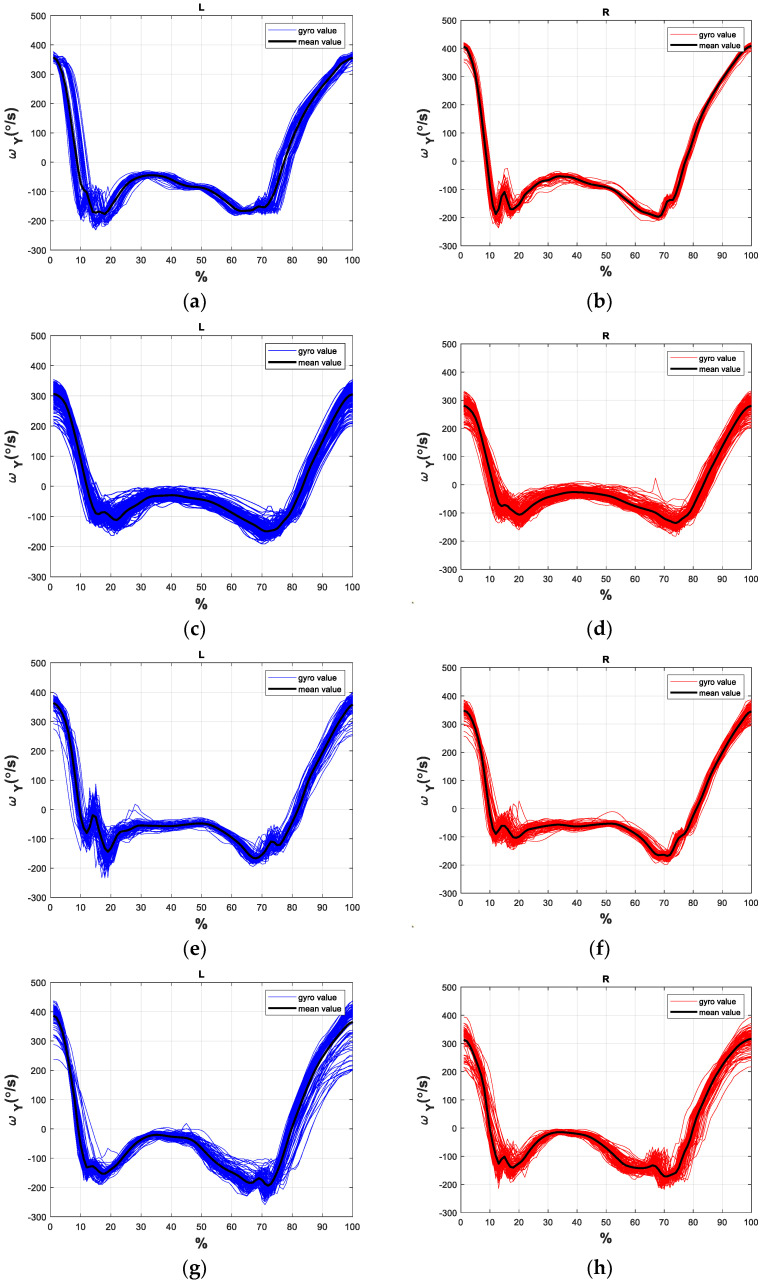

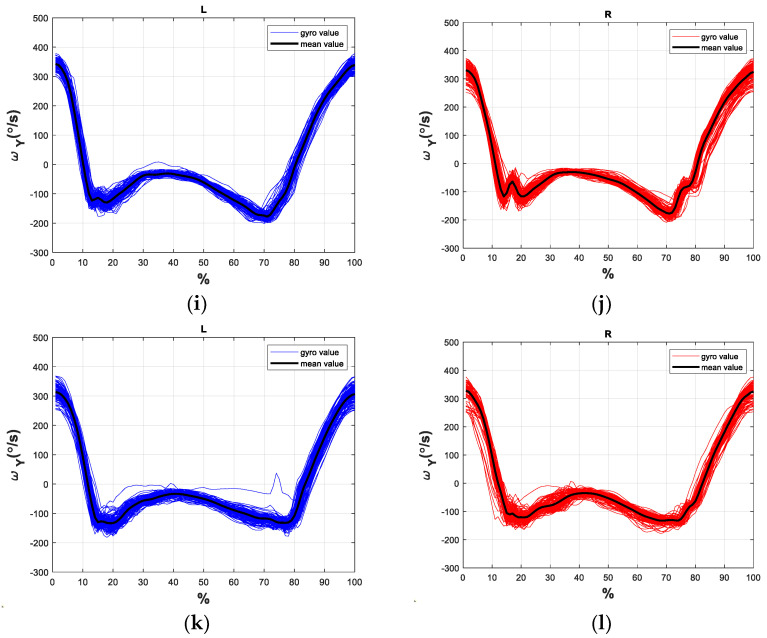
The gait patterns of healthy subjects. (**a**) left gait cycles of H1; (**b**) right gait cycles of H1; (**c**) left gait cycles of H2; (**d**) right gait cycles of H2; (**e**) left gait cycles of H3; (**f**) right gait cycles of H3; (**g**) left gait cycles of H4; (**h**) right gait cycles of H4; (**i**) left gait cycles of H5; (**j**) right gait cycles of H5; (**k**) left gait cycles of H6; (**l**) right gait cycles of H6.

## Data Availability

The dataset of gaits applied in this paper is available at http://140.112.14.7/~sic/PaperMaterial/Dataset.zip. (accessed on 1 March 2021) The public dataset PAMAP2 is available at: http://archive.ics.uci.edu/ml/datasets/PAMAP2+Physical+Activity+Monitoring. (accessed on 1 March 2021).

## Appendix A

The studies were approved by the Human Subject Research Ethics Committee of the Institutional Review Board (IRB), available at: http://140.112.14.7/~sic/PaperMaterial/IRB_renew.pdf. (accessed on 1 March 2021).

## Appendix B

The gait cycles of the stroke patients P1~P7 and the healthy subjects H1~H6.

## Appendix C

The dataset of gaits in this paper is available at: http://140.112.14.7/~sic/PaperMaterial/Dataset.zip. (accessed on 1 March 2020).

## Appendix D

The data of two stroke subjects for model verification.

**Table A1:** Basic data of the two stroke subjects for model verification.

Stroke Subject								
Subject	Gender	Age	Height (cm)	Weight (kg)	Paretic Side	MMSE (Score)	BS (Stage)	FAC (Stage)
Sv1	Male	41	171	70	left	30	4	6
Sv2	Female	50	155	52	left	30	3	6
